# Does weight loss reduce the incidence of total knee and hip replacement for osteoarthritis?—A prospective cohort study among middle-aged and older adults with overweight or obesity

**DOI:** 10.1038/s41366-021-00832-3

**Published:** 2021-05-15

**Authors:** Xingzhong Jin, Alice A. Gibson, Joanne Gale, Francisco Schneuer, Ding Ding, Lyn March, Amanda Sainsbury, Natasha Nassar

**Affiliations:** 1 Centre for Big Data Research in Health, The University of New South Wales, Sydney, Australia; 2 Institute of Bone and Joint Research, Kolling Institute, The University of Sydney, Sydney, Australia; 3 The Boden Collaboration for Obesity, Nutrition, Exercise & Eating Disorders, , The University of Sydney, Sydney, Australia; 4 Menzies Centre for Health Policy, Sydney School of Public Health, Faculty of Medicine and Health, The University of Sydney, Sydney, Australia; 5 Sydney School of Public Health, The University of Sydney, Sydney, Australia; 6 Child Population and Translational Health, The Children’s Hospital at Westmead Clinical School, The University of Sydney, Sydney, Australia; 7 School of Human Sciences, The University of Western Australia, Perth, Australia

**Keywords:** Epidemiology, Epidemiology

## Abstract

**Objective:**

This study aims to investigate the association between weight change and total knee or hip replacement (TKR or THR) for OA among middle-aged and older adults with overweight or obesity.

**Method:**

Weight data were collected in 2006–2009 and in 2010 from the 45 and Up Study—a population-based cohort aged ≥45 years in New South Wales, Australia. Participants were included if they had a baseline body mass index (BMI) ≥ 25 kg/m^2^ and no history of TKR or THR. Weight change was categorised into four groups: >7.5% loss; >5–7.5% loss; stable (≤5% change) and >5% gain. Hospital admission data were linked to identify TKR and THR for OA, and multivariable Cox regression was used to assess risk of TKR and THR.

**Results:**

Of 23,916 participants, 2139 lost >7.5% weight, 1655 lost 5–7.5% weight, and 4430 gained >5% weight. Over 5.2 years, 1009 (4.2%) underwent TKR and 483 (2.0%) THR. Compared to weight-stable, weight loss of >7.5% was associated with reduced risk of TKR after adjusting for age, sex, BMI, socioeconomic and lifestyle factors (hazard ratio 0.69, 95%CI 0.54–0.87), but had no association with THR. Weight loss of 5–7.5% was not associated with altered risk of either TKR or THR. Weight gain was associated with increased risk of THR after adjusting for confounders, but not TKR.

**Conclusion:**

This study suggests that a weight loss target >7.5% is required to reduce the risk of TKR in adults with overweight or obesity. Weight gain should be avoided as it increases the risk of THR.

## Introduction

Osteoarthritis (OA) is a public health problem and was ranked as the 11th highest contributor (out of 310 diseases) to global disability in 2015 [1]. Large weight-bearing joints, notably the knee and hip, are usually affected by the disease in people aged 45 and over [2]. Presently, there are no approved pharmacotherapies that prevent or halt the progression of OA. For severe knee or hip OA, the ‘last resort’ treatments are total knee or hip replacement surgery (TKR or THR). The global economic burden of TKR and THR due to OA is high [3], and is predicted to increase substantially in the next two decades in many countries, including the United States [4], United Kingdom [5], Canada [6], Sweden [7] and New Zealand [8]. In Australia, the healthcare costs for OA were over $2.1 billion in 2015, and 77% of these costs were attributable to admitted hospital costs for TKR or THR for OA [9]. By the year 2030, the incidence of TKR and THR in Australia is expected to rise by 276% and 208%, respectively [10]. Similar projections were also reported for other countries [4, 5] due to an aging world population and the increasing prevalence of obesity [11].

Obesity is a well-recognised risk factor for the development and progression of OA, and likely contributes to the need for total joint replacement [12]. A recent meta-analysis found that people with obesity had a 4.6-fold greater risk of developing OA compared to people with healthy weight [13]. People with excess weight are more likely to progress to an advanced stage of OA, with estimates suggesting that 80–95% of people needing a total joint replacement also had overweight or obesity [14, 15]. Considering the strong link between obesity and increased risk of OA as well as total joint replacement, all clinical guidelines for OA management around the world recommend weight loss as a core strategy for all OA patients with overweight and obesity [16–20]. However, few studies have assessed and quantified the clinical benefits of weight loss for OA. A systematic review and meta-analysis of four randomised controlled trials showed a statistically significant effect of weight loss to reduce pain and disability in people with knee OA, but it was unclear whether these statistically significant changes were of sufficient magnitude to be clinically significant [21]. Indeed, the meta-analysis suggested that a loss of 5% of initial weight within a 20-week period is required to obtain moderate symptomatic relief [21]. In contrast to these studies on knee OA, there are no studies investigating the specific effects of weight loss on hip OA. More importantly, for both knee and hip OA, it remains unclear whether weight loss reduces the risk for TKR and THR in the long term. Therefore, the objective of this longitudinal study was to investigate the association of weight loss recommended by current guidelines and the risk of TKR and THR due to OA among middle-aged and older adults with overweight or obesity.

## Method

### Study design and data sources

**Fig. 1:**
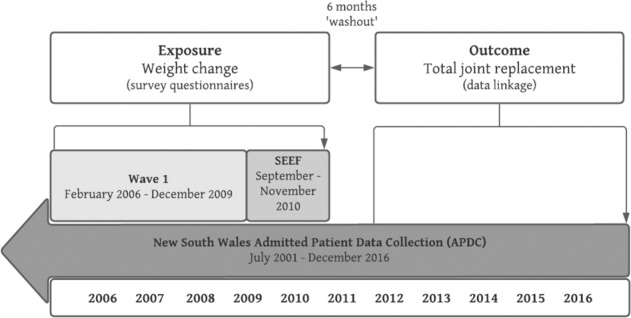
Study design schema.

This study used data from the Sax Institute’s 45 and Up Study, which is a large longitudinal study of healthy ageing in the general population living in New South Wales (NSW), Australia [22]. The research protocol has been published in detail previously [23], and is described briefly here. Prospective participants were randomly sampled from the enrolment database of the Department of Human Services, which provides near-complete coverage of the Australian population. People living in rural areas and those aged 80 years and over were oversampled to provide clear statistical pictures of these groups. Baseline data (Wave 1) were collected via self-report surveys between February 2006 and December 2009 from 267,153 individuals aged ≥45 years (Fig. 1). These participants provided informed consent for subsequent follow-up surveys and access to their data from administrative health records via data linkage. Following baseline survey, the first 100,000 participants were contacted for a second survey in September to November 2010, as part of a separate study, the Social Economic and Environmental Factors (SEEF) Study, and were included in our study so we could measure a longitudinal change in weight (Fig. 1).

For the current study, the SEEF study participants were followed up for outcome data collection via probabilistic data linkage to their corresponding administrative hospital and death records. These were obtained from the NSW Admitted Patient Data Collection (APDC, July 2001 to December 2016) and the NSW Registry of Births, Death and Marriages (January 2006 to December 2016), respectively (Fig. 1). Data linkage was conducted by the NSW Centre for Health Record Linkage.

### Ethics committee approval

Ethics approval for the 45 and Up study was granted by the University of NSW Human Research Ethics Committee, and approval for the current study was obtained from the NSW Population Health Services Research Ethics Committee.

### Patient and public involvement (PPI)

We did not directly include PPI in this study, but the database used in the study was developed with PPI and is updated by a committee that includes patient representatives.

### Study exposure, outcomes and confounders

Our exposure of interest was the change in weight from the baseline (Wave 1) to follow-up (SEEF) surveys. The weight data were self-reported at both timepoints. Weight change was categorised into four groups: weight loss >7.5%; weight loss >5–7.5%; weight-stable (≤5% change), and weight gain >5%. We used 5% and 7.5% as cut-offs for weight loss because current guidelines from the Osteoarthritis Research Society International [16] and the Royal Australian College of General Practitioners [20] recommend a weight loss target of 5% and 5–7.5% of initial weight, respectively, for effective management of OA in people with overweight or obesity.

Study outcomes were defined as the incidence of TKR or THR for OA and determined using the linked hospital admission records from the NSW Admitted Patient Data Collection (APDC, Fig. 1). The APDC is a statutory state-wide, administrative data collection of all public and private hospital in-patient admissions in NSW, Australia. The APDC data were linked to the 45 and Up Study using probabilistic matching conducted by the NSW Centre for Health Record Linkage (CHeReL) with a false positive rate of 0.5%. Cases of TKR and THR were identified according to the codes used by the Australian National Joint Replacement Registry [24], which include a procedure code for TKR or THR based on the Australian Classification of Healthcare Interventions (ACHI) procedural codes, together with a primary diagnosis code for knee OA or hip OA based on the International Classification of Diseases, 10th Revision, Australian Modification (ICD10-AM). If a participant had multiple knee (or hip) replacements during the follow-up, the first replacement surgery of the knee (or hip) was used to defined as an incidence.

We selected a list of confounders based on background knowledge and prior literature evidence. Potential confounders selected for this study were age, sex, body mass index (BMI), education level [25], private health insurance status [26], smoking status [27], physical activity level [28], and self-reported OA status (“In the last month have you been treated for osteoarthritis”). Information of these confounders were based on self-reported responses at the baseline survey (Wave 1). BMI was derived from body weight in kilograms divided by the square of the body height in metres. Physical activity level was assessed using the Active Australia Survey [29]. The number of minutes spent on physical activity per week was calculated as the sum of the number of minutes spent on three types of activity, namely walking (generally considered as low-intensity), moderate-intensity, and vigorous-intensity physical activity, with the minutes of vigorous-intensity physical activity weighted by 2 according to the Active Australia Protocol [29]. Based on the minutes of physical activity per week, participants were classified into one of three physical activity levels: <150 min (inactive or insufficiently active); 150 to 299 min (sufficiently active); or ≥300 min (highly active). This classification is similar to the Australian’s Physical Activity and Sedentary Behaviour Guidelines [30], except that minutes of walking is not included in the government guidelines.

### Study participants

Participants were included in the present study if they had: valid weight data collected in both the 45 and Up Study (baseline Wave 1) and SEEF Study (follow-up), a BMI ≥ 25 kg/m^2^ and no self-reported diagnosis of cancer at baseline (because cancer is often associated with unintentional weight loss). Participants who had a TKR (or THR) before 6 months following the completion of the SEEF Study survey were further excluded from the TKR (or THR) analysis sample. This exclusion procedure was conducted because patients undergoing elective TKR (or THR) are routinely advised to lose weight 6 months prior to surgery if they have overweight or obesity [31], and including these participants in our study would bias the analysis toward a positive association between weight loss and total joint replacement.

### Statistical analysis

Means and standard deviations (for continuous variables) and percentages (for categorical variables) were used to describe the baseline characteristics of participants, grouped by weight change categories. The associations between weight change and the risk for TKR and THR were each analysed using Cox proportional hazards regression models and hazard ratios (HRs), and their 95% confidence intervals (CIs) were calculated. Weight-stable (≤5% change) was used as the reference group for comparison. Following univariable analysis, multivariable analyses were conducted, first adjusting for age, sex and baseline BMI (Model 1), and then additionally adjusting for education level, private health insurance status, smoking status, physical activity level, and OA status (Model 2). Given the impact OA status may have on findings, we examined the potential interaction between OA status and weight change. We also conducted sensitivity analyses using percentage of weight loss as continuous variable to explore the linear relationship with the risk of TKR and THR. All analyses were performed using SAS software version 9.4 (SAS Institute Inc., Cary, North Carolina), and statistical significance was defined as a two-tailed *p*-value < 0.05.

## Results

**Fig. 2:**
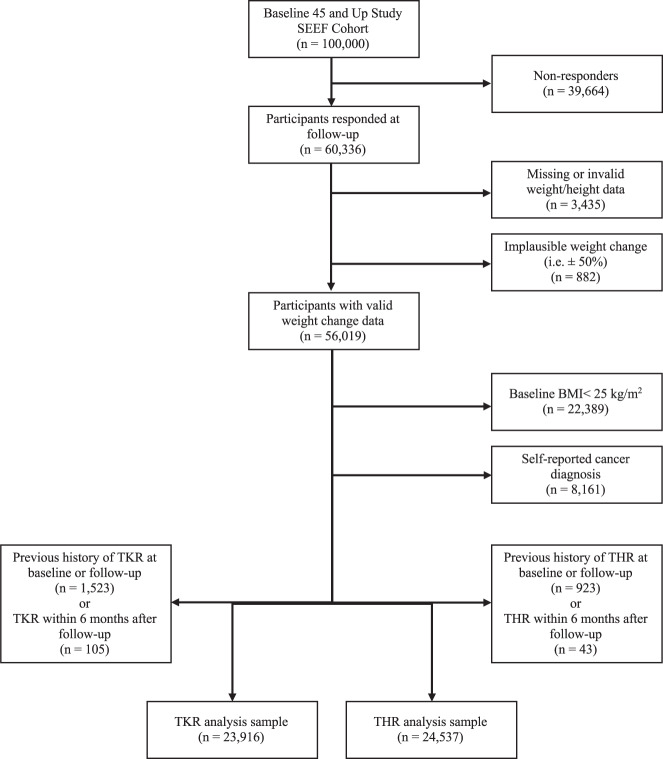
Study sampling flowchart.

A flowchart of participants in this study is presented in Fig. 2. A total of 60,336 participants responded to the follow-up survey (response rate 60.3%). The characteristics of the participants who responded were comparable to the original 45 and Up Study cohort in terms of sex, BMI and OA status (Supplementary Table 1). After exclusion of ineligible participants, a total of 25,469 participants were available for this study (Fig. 2). Of these, 23,916 and 24,537 participants were included in the analysis of TKR and THR for OA, respectively. Table 1 presents the baseline characteristics of the cohort included in the analysis of TKR for OA. The baseline characteristics of the cohort included in the analysis of THR for OA were similar (Supplementary Table 2). The four weight change groups were similar in terms of age, sex, BMI and other potential confounders selected for this study.

**Table 1:** Baseline characteristics and knee replacement rate of participants in the total knee replacement for osteoarthritis analysis.

	Weight change from baseline to follow-up				Total
	Loss > 7.5%	Loss 5–7.5%	Stable	Gain > 5%	
*n*	2139	1655	15,692	4430	23,916
Age (years)	61.5 ± 10.7	62 ± 10.2	60.6 ± 9.4	57.7 ± 8.4	60.2 ± 9.5
Female (%)	1260 (58.9)	837 (50.6)	7127 (45.4)	2605 (58.8)	11,829 (49.5)
Body mass index (kg/m^2^)	31.3 ± 5.8	29.7 ± 4.3	29.2 ± 3.9	29.5 ± 4.2	29.5 ± 4.3
Treated for OA in the last month (%)	331 (15.5)	232 (14.0)	1831 (11.7)	544 (12.3)	2938 (12.3)
Education					
Up to School or Intermediate Certificate	750 (35.1)	518 (31.3)	4614 (29.4)	1406 (31.7)	7288 (30.5)
Higher School to Diploma	846 (39.6)	718 (43.4)	6806 (43.4)	1929 (43.5)	10,299 (43.1)
Degree of higher	519 (24.3)	397 (24.0)	4105 (26.2)	1043 (23.5)	6064 (25.4)
Have private health insurance (%)	1346 (62.9)	1085 (65.6)	10,858 (69.2)	2897 (65.4)	16,186 (67.7)
Physical activity level (%)					
<150 min per week	518 (24.2)	331 (20.0)	2978 (19.0)	919 (20.7)	4746 (19.8)
150–299 min per week	358 (16.7)	264 (16.0)	2667 (17.0)	763 (17.2)	4052 (16.9)
300+ minutes per week	1193 (55.8)	1003 (60.6)	9699 (61.8)	2657 (60.0)	14,552 (60.8)
Smoking status (%)					
Non-smoker	1197 (56.0)	903 (54.6)	9123 (58.1)	2388 (53.9)	13,611 (56.9)
Ex-smoker	786 (36.7)	667 (40.3)	5837 (37.2)	1649 (37.2)	8939 (37.4)
Current smoker	156 (7.3)	85 (5.1)	731 (4.7)	393 (8.9)	1365 (5.7)

*OA,* osteoarthritis.

Numbers may not add up to totals due to missing data.

### Total knee replacement for osteoarthritis

**Fig. 3:**
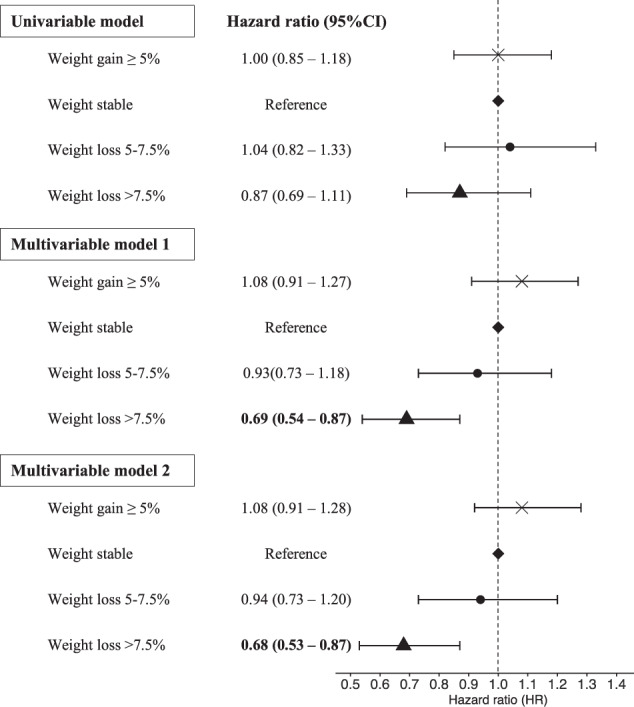
Association between weight change and incidence of total knee replacement.

For the analyses of TKR for OA, most participants (65.6%) maintained a stable weight (≤5% change), while 2,139 (8.9%) lost >7.5% weight, 1,655 (6.9%) lost >5–7.5% weight, and 4,430 (18.5%) gained >5% weight (Table 1). The mean follow-up time for the TKR analysis was 5.2 years (range 4.7–6.0 years), and a total of 1,009 (4.2%) underwent TKR for OA during this follow-up period. Compared to those with a stable weight, individuals with weight loss >7.5% had a reduced risk of TKR (HR 0.69, 95%CI 0.54–0.87) after adjusting for age, sex and baseline BMI (Multivariable Model 1, Fig. 3). There was little change in the results after further adjusting for education level, private health insurance status, smoking status, physical activity level and OA status (HR 0.68, 95%CI 0.53–0.87, Multivariable Model 2, Fig. 3). Neither >5–7.5% weight loss nor >5% weight gain was significantly associated with altered risk of TKR. There was also no significant interaction between OA status and weight change (*p* = 0.70). In the sensitivity analysis, a linear association was observed between percentage of weight loss and risk of TKR (HR 1.02, 95%CI 1.01–1.03). In other words, for every 1% reduction in weight, there was a 2% reduction in risk of TKR.

### Total hip replacement for osteoarthritis

**Fig. 4:**
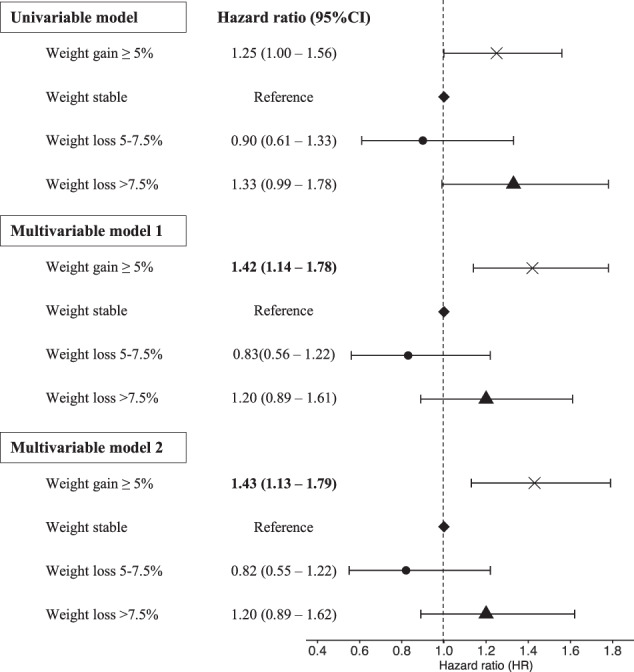
Association between weight change and incidence of total hip replacement.

The mean follow-up time between exposure and outcome data collection for this cohort was 5.2 years (range 4.7–6.0 years), and 483 (2.0%) had THR for OA during this follow-up period. Similar to the TKR analysis, the majority of participants (65.5%) maintained a stable weight (≤5% change), while 2,222 (9.1%) lost >7.5% weight, 1,692 (6.9%) lost >5–7.5% weight, and 4,562 (18.6%) gained >5% weight. In contrast to TKR, those who lost >7.5% weight did not appear to have a significantly increased risk of THR for OA compared to the weight-stable reference group (Fig. 4). However, those who gained >5% weight had an increased risk of THR for OA, with an HR of 1.25 (95%CI 1.00–1.56) (Fig. 4). The association was borderline significant, but became stronger after adjusting for confounders (Model 1 and Model 2, Fig. 4). There was no interaction between OA status and weight change (*p* > 0.05). The linear association between percentage of weight change and risk of THR was not statistically significant (HR 1.01, 95%CI 1.00–1.03).

## Discussion

This study provides circumstantial evidence that middle-aged and older adults with overweight or obesity who lost >7.5% of their initial weight had reduced risk of TKR for OA compared to those with a stable weight (≤5% change). Neither losing >5–7.5% of weight nor gaining >5% weight significantly impacted the risk of TKR. Although losing weight was not associated with the risk of THR, gaining weight increased the risk of THR for OA.

Our results extend previous research which highlighted the benefits of weight loss on knee OA symptoms. Evidence from a systematic review and meta-analysis suggested that weight loss was likely to reduce symptoms of OA and improve joint function in individuals with overweight and knee OA [21]. The effect of weight loss on symptom reduction in knee OA appears to have a dose-response relationship [32]. Specifically, weight loss of at least 7.7% was required to achieve a clinically meaningful improvement in pain and physical function in patients with knee OA and concurrent overweight or obesity [32]. The findings from our analyses show a similar trend between weight loss and reduction in TKR for OA, and suggest that a weight loss of at least 7.5% is required to reduce TKR for OA. Furthermore, we conducted an exploratory analysis stratified by overweight and obesity subgroups. The results showed that while the association between weight loss >7.5% and reduced TKR risk remained and was statistically significant in the overweight subgroup, there was no association in the obesity subgroup, suggesting a higher percentage of weight loss is required for the obesity group.

It is worth noting that the lack of statistical significance in the association between weight gain and TKR could be due to a statistical ‘ceiling effect’—i.e., the variance of weight change was no longer measurable when participants were grouped into the weight gain category. To explore this ceiling effect of weight gain, we conducted a subgroup analysis that used percentage weight loss as a continuous variable and examine the linear association separately in participants who lost and gained weight. The analysis showed a significant linear association between increasing percentage of weight change and TKR in both weight gain (HR 1.03, 95%CI 1.01–1.04) and weight-loss (HR 1.04, 95%CI 1.02–1.06) subgroups. Although the elevated HR for TKR in the weight gain >5% group in our primary analysis did not reach a statistical significance when comparing to the weight stable group, people with overweight or obesity should still avoid further weight gain for the benefits of reducing the risk of cardiovascular comorbidities and diabetes [33].

The current study addresses the knowledge gap as to whether or not weight loss slows the progression of knee OA that leads to joint replacement surgery [34]. One study in a cohort of people with obesity showed that weight loss was associated with reduced medial cartilage volume loss in the knees over 2.3 years, suggesting a disease-modifying effect of weight loss on knee joint structure [35]. Data from the Osteoarthritis Initiative also found that an appreciable weight loss (>10% of initial weight) slowed knee cartilage degeneration in people with overweight and obesity over a period of 4 years [36]. In contrast, a randomised controlled trial reported that no differences were observed in cartilage loss, bone marrow lesion or synovitis over 1.5 years, between people with OA who lost ≥10% of their initial weight compared to those who did not [37]. Similarly, in a more recent study among individuals with severe obesity undergoing either bariatric surgery, pharmacotherapy or other non-surgical medical interventions for weight loss, participants who lost ≥20% of their initial body weight had a rapid reduction in pain from knee OA over 1 year, but this was not accompanied by structural changes in the knee compared to those who lost <20% of their initial weight [38]. The results from our study address this knowledge gap by showing the long-term benefit of weight loss >7.5% in reducing progression to TKR for OA in individuals with overweight or obesity.

Although weight loss is recommended for patients with hip OA by most OA guidelines [17, 18, 20], evidence of its benefits on hip OA symptoms and THR is lacking. In the latest OARSI guidelines for non-surgical management for hip OA, dietary weight management was not recommended ‘because of lack of direct evidence for its effectiveness specifically for symptoms of hip OA’ [39]. One prospective cohort study reported that weight loss in combination with exercise could reduce pain from hip OA and increase hip physical function after 8 months [40], while another prospective cohort study reported that overweight was not a determinant of the onset or progression of hip radiographic OA [41]. Our results were in agreement with—and extend—the latter study, suggesting that weight loss was not associated with reduced risk of THR for OA. The null association may be a result of the hip joint being less sensitive to obesity and weight change than the knee joint [41, 42]. It is possible that the reduction in mechanical loading of the joint resulting from weight loss is different in the knee and the hip. The knee and the hip have different anatomy: the knee joint is a hinge joint, whereas the hip is a ball-and-socket joint. Changes in force on a misaligned hinge joint (e.g., an OA-affected knee joint) may be magnified by two to three times compared with a normal hinge joint, due to the small area that the forces act on [41]. In contrast, a misaligned ball-and-socket joint (e.g., an OA-affected hip joint) might be less sensitive to changes in force, as they are distributed over a larger area compared to the knee. These findings do not negate the notion that individuals with overweight or obesity should avoid further weight gain, which was shown to significantly increase the risk of THR for OA in the present study.

### Strengths and limitations

To our knowledge, the present study is the first prospective cohort study to investigate the association of weight loss on the reduction in TKR and THR for OA. A major strength of our study design is that, instead of analysing a cross-sectional correlation between current weight and joint replacement surgery, we observed the long-term incidence of TKR and THR for OA after weight change had occurred. Other strengths of this study include the large sample size from a population-based cohort (over 60,000 people), and the use of data linkage to state-wide hospital and death records to ascertain outcomes. We also acknowledge that there are limitations inherent to this study. One limitation is that OA at baseline was defined by self-reported treatment for OA, and we did not have information on whether OA affects the knees, the hips or both, which may have introduced recall bias or misclassification bias [43]. Therefore, we included both OA and non-OA population in the main analysis in an effort to mitigate the impact of these biases. This also extends the generalisability of the results to a broader population with overweight or obesity. Another limitation is that our analyses were not able to explore the confounding effect of important risk factors for receiving TKR and THR, such as the previous history of joint injury, or disease severity of OA measured by X-ray and joint pain, because these data were not recorded in this population cohort study. Also, our data did not record details about how the weight loss was achieved (e.g. via dietary treatment, physical exercise, or bariatric surgery), it is possible that different means of weight loss may influence the risk of TKR and THR differently due to various physiological mechanisms. Lastly, we were not able to identify the side of the body on which the TKR or THR was performed, because the ICD10-AM codes used to report TKR or THR do not specify sidedness. As a result, we excluded participants who self-reported having had any previous TKR or THR at the start of follow-up, in order to reduce contamination by repeated TKR or THR. However, we do not see any reason why the observed effects of weight loss would not also be applicable to the opposite side of the body, for people who have already had a TKR or THR on one side of the body. National joint replacement registries, such as the Australian Orthopaedic Association National Joint Replacement Registry (AOANJRR), usually have laterality information recorded. Future studies that have the data linkage capacity with a national joint replacement registry will be able to confirm the effects of weight loss on the risk of contralateral joint replacement in people with one side of the joints replaced.

Even though there are very few contraindications to TKR and THR and the mortality rates following the procedures have decreased substantially over recent decades, there are serious adverse events associated with both of these procedures, including sepsis, pulmonary embolism and cardiac complications [44]. Besides, approximately 20% and 9% of patients experience persistent joint pain 12 months after TKR and THR, respectively [45]. Therefore, joint replacement treatment should be reserved as the last treatment option for those with end-stage OA when conservative management has failed [20]. Setting an evidence-based weight loss target is important for effectively managing knee and hip OA to reduce or delay the need for joint replacement surgery. While weight loss is recommended as a core management strategy in OA clinical management guidelines around the world, few of them highlights a necessity for weight loss of over 7.5%. For example, the Osteoarthritis Research Society International recommends a weight loss of 5% to be efficacious [16], while the Royal Australian College of General Practitioners recommend a minimum weight loss target of 5–7.5% for all patients with OA and overweight or obesity [20]. The present study suggests a weight loss target of >7.5% to reduce the risk of TKR for OA. Future studies are needed to address the limitations of this study. In particular, the weight loss target >7.5% should be further confirmed in clinical trials or in long-term epidemiology studies in an OA population, such as the Osteoarthritis Initiative [46].

## Conclusion

A weight loss of >7.5% is associated with a reduced risk of TKR for OA among middle-aged and older adults with overweight or obesity. Further weight gain should be avoided to prevent an increased risk of THR for OA.

### Data sharing statement

This research was completed using data collected from the Sax Institute’s 45 and Up Study. Requests for access to data should be addressed to the corresponding author or the Sax Institute (http://www.saxinstitute.org.au/).

## Supplementary information

Supplementary materials
