# Supplementary material for: Does weight loss reduce the incidence of total knee and hip replacement for osteoarthritis?—A prospective cohort study among middle-aged and older adults with overweight or obesity
Source: Int J Obes (Lond). 2021 May 15;45(8):1696–704. doi: 10.1038/s41366-021-00832-3 (PMC8310800; doi:10.1038/s41366-021-00832-3)
Supplement: Supplementary file 1 — Supplementary materials [file 41366_2021_832_MOESM1_ESM.docx]

| **Supplementary Table 1. Characteristics of participants in the SEEF Study and the original 45 and Up Study cohort.** | | |
| --- | --- | --- |
|  | **SEEF Study^*^** | **45 and Up Study** |
|  | **N (%)** | **N (%)** |
| **Body mass index (kg/m^2^)** | | |
| < 18.5 | 700 (1.9) | 3,408 (1.4) |
| 18.5 - 24.9 | 13,342 (35.8) | 90,794 (36.8) |
| 25.0 - 29.9 | 14,241 (38.3) | 97,441 (39.5) |
| ≥ 30.0 | 8,936 (24.0) | 55,054 (22.3) |
| **Sex** | | |
| Male | 28,023 (46.4) | 123,806 (46.4) |
| Female | 32,313 (53.6) | 143,035 (53.6) |
| **Treated for OA in the last month** | | |
| No | 53,957 (89.4) | 245,263 (91.9) |
| Yes | 6,379 (10.6) | 21,578 (8.1) |
| **Total** | 60,336 (100) | 266,841 (100) |
| *^*^The first 100,000 participants from the 45 and Up Study were contacted to take part in the Social Economic and Environmental Factors (SEEF) Study,* | | |

| **Supplementary Table 2. Baseline characteristics of study participants of the hip replacement for osteoarthritis analysis** | | | | | |
| --- | --- | --- | --- | --- | --- |
|  | **Weight change from baseline to follow-up** | | | | **Total** |
|  | **Loss >7.5%** | **Loss 5-7.5%** | **Stable** | **Gain > 5%** |  |
| **n** | 2,222 | 1,692 | 16,061 | 4,562 | 24,537 |
| **Age (years)** | 61.6 ± 10.6 | 62.1 ± 10.2 | 60.8 ± 9.4 | 58.0 ± 8.6 | 60.4 ± 9.5 |
| **Female (%)** | 1,315 (59.2) | 858 (50.7) | 7,327 (45.6) | 2,703 (59.3) | 12,203 (49.7) |
| **Body mass index (kg/m^2^)** | 31.4 ± 5.8 | 29.8 ± 4.3 | 29.3 ± 4 | 29.6 ± 4.2 | 29.6 ± 4.3 |
| **Treated for OA in the last month (%)** | 368 (16.6) | 248 (14.7) | 1,972 (12.3) | 603 (13.2) | 3,191 (13.0) |
| **Education** |  |  |  |  |  |
| Up to School or Intermediate Certificate | 779 (35.1) | 530 (31.3) | 4,810 (29.9) | 1,475 (32.3) | 7,594 (30.9) |
| Higher School to Diploma | 881 (39.6) | 727 (43.0) | 6,931 (43.2) | 1,984 (43.5) | 10,523 (42.9) |
| Degree or higher | 538 (24.2) | 412 (24.3) | 4148 (25.8) | 1,047 (23.0) | 6,145 (25.0) |
| **Have private health insurance (%)** | 1,399 (63.0) | 1,117 (66.0) | 11,094 (69.1) | 2,982 (65.4) | 16,592 (67.6) |
| **Physical activity per week (%)** |  |  |  |  |  |
| <150 minutes | 554 (24.9) | 341 (20.2) | 3,079 (19.2) | 961 (21.1) | 4,935 (20.1) |
| 150 - 299 minutes | 370 (16.7) | 276 (16.3) | 2,718 (16.9) | 788 (17.3) | 4,152 (16.9) |
| 300+ minutes | 1,234 (55.5) | 1,016 (60) | 9,901 (61.6) | 2,714 (59.5) | 14,865 (60.6) |
| **Smoking status (%)** |  |  |  |  |  |
| Non-smoker | 1,245 (56.0) | 925 (54.7) | 9,354 (58.2) | 2,477 (54.3) | 14,001 (57.1) |
| Ex-smoker | 821 (36.9) | 681 (40.2) | 5,974 (37.2) | 1,691 (37.1) | 9,167 (37.4) |
| Current smoker | 156 (7.0) | 86 (5.1) | 732 (4.6) | 394 (8.6) | 1,368 (5.6) |
| *OA, osteoarthritis. Numbers may not add up to totals due to missing data.* | | | | | |
